# Intratumoural Cytochrome P450 Expression in Breast Cancer: Impact on Standard of Care Treatment and New Efforts to Develop Tumour-Selective Therapies

**DOI:** 10.3390/biomedicines9030290

**Published:** 2021-03-12

**Authors:** Smarakan Sneha, Simon C. Baker, Andrew Green, Sarah Storr, Radhika Aiyappa, Stewart Martin, Klaus Pors

**Affiliations:** 1 Institute of Cancer Therapeutics, School of Pharmacy and Medical Sciences, Faculty of Life Sciences, University of Bradford, Bradford BD7 1DP, UK; S.Swaroop@bradford.ac.uk; 2 Jack Birch Unit for Molecular Carcinogenesis, Department of Biology & York Biomedical Research Institute, University of York, Heslington, York YO10 5DD, UK; simon.baker@york.ac.uk; 3 Nottingham Breast Cancer Research Centre, School of Medicine, Biodiscovery Institute, University Park, Nottingham NG7 2RD, UK; andrew.green@nottingham.ac.uk (A.G.); sarah.storr@nottingham.ac.uk (S.S.); Radhika.Aiyappa@nottingham.ac.uk (R.A.); stewart.martin@nottingham.ac.uk (S.M.)

**Keywords:** Cytochrome P450, CYP1A1, CYP1B1, CYP2W1, breast cancer, prodrug, bioprecursor, duocarmycin, phortress, AQ4N

## Abstract

Despite significant advances in treatment strategies over the past decade, selective treatment of breast cancer with limited side-effects still remains a great challenge. The cytochrome P450 (CYP) family of enzymes contribute to cancer cell proliferation, cell signaling and drug metabolism with implications for treatment outcomes. A clearer understanding of CYP expression is important in the pathogenesis of breast cancer as several isoforms play critical roles in metabolising steroid hormones and xenobiotics that contribute to the genesis of breast cancer. The purpose of this review is to provide an update on how the presence of CYPs impacts on standard of care (SoC) drugs used to treat breast cancer as well as discuss opportunities to exploit CYP expression for therapeutic intervention. Finally, we provide our thoughts on future work in CYP research with the aim of supporting ongoing efforts to develop drugs with improved therapeutic index for patient benefit.

## 1. Introduction

As our understanding of cancer improves, it has become increasingly evident that targeted and patient-specific treatment strategies are essential for effective treatment. A one-size-fits-all approach has multiple flaws in the face of a wide range of cancer subtypes, with each patient harbouring specific genetic alterations and metabolic profiles. Immunohistochemical studies have classified breast cancer into four broad subtypes: oestrogen receptor-positive (ER+), progesterone receptor-positive (PR+), human epidermal growth factor receptor 2-positive (HER2+) and triple negative [1]. Patients harbouring tumours that do not express any ER, PR or HER2 receptors are classified as triple negative breast cancers (TNBC) [2]. Around 80% of the breast tumours are positive for the hormone receptors wherein binding of the requisite chemical messenger to their receptor causes changes in the cancer cell resulting in proliferation [3]. Although these receptors can be targeted by drugs that disrupt the signalling process, hormonal therapies are still associated with undesirable side effects including bone weakness and temporary or permanent menopausal symptoms [4].

TNBC is the least common of all subtypes, disproportionately affecting pre-menopausal African American women [5] and has been associated with poor clinical outcome. Treatment relies heavily on chemotherapy as endocrine and targeted (e.g., trastuzumab-based) therapies are of no benefit in this disease [6]. As a consequence, the number of drug combinations that can be used are limited thereby increasing the risk of developing drug resistance [7]. Furthermore, there is far less understanding of how to treat TNBC compared to receptor-positive cases as there are currently no known biomarkers to classify TNBCs according to response. Further research is required to unravel the underlying causes of TNBC, with deficiencies in underlying DNA repair mechanisms often being implicated [8,9,10].

## 2. Cytochrome P450 Function

The cytochrome P450 (CYP) superfamily of enzymes consist of a group of 57 human isoforms that have the capacity to oxidise a vast variety of endogenous and xenobiotic compounds [11]. In mammals, these enzymes are primarily involved in the metabolism of drugs and fat-soluble vitamins, conversion of polyunsaturated fatty acids to biologically active compounds and in the synthesis of steroid hormones. Apart from the capacity to detoxify and inactivate many clinically used drugs, CYPs are also involved in conversion of prodrugs into cytotoxic metabolites [12]. The ability of these enzymes to metabolise both procarcinogens and anticancer drugs makes their study interesting in terms of cancer aetiology and therapy. Differential endogenous expression of certain CYP genes in tumour vs. normal tissue provides an opportunity for selective targeting with small molecules designed to capitalise on CYP catalysis [13,14].

## 3. CYPs in Breast Cancer

**Figure 1:**
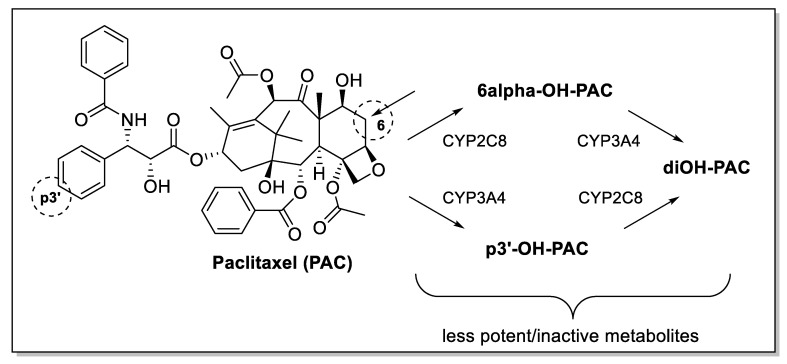
CYP metabolism contributes to paclitaxel treatment failure.

**Figure 2:**
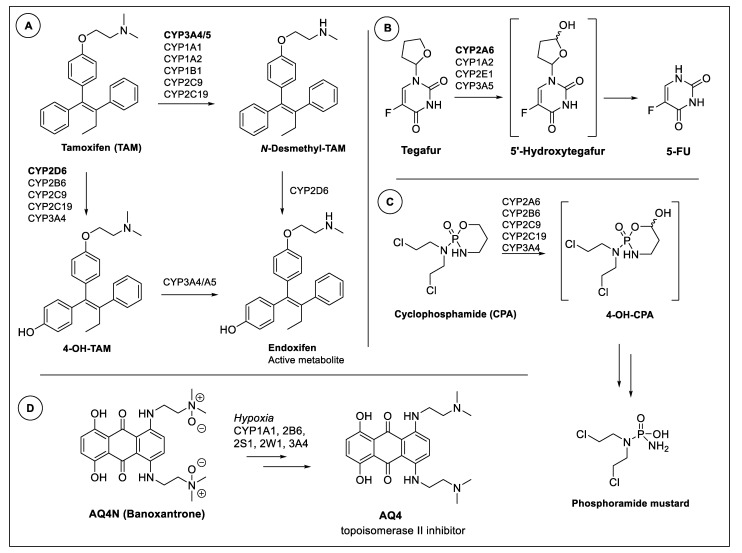
Non-selective bioactivation of prodrugs tamoxifen (**A**), tegafur (**B**), CPA (**C**) and tumour-selective hypoxia-activated prodrug AQ4N (**D**).

A clearer understanding of CYP expression is important in the pathogenesis of breast cancer as several isoforms play critical roles in metabolising steroid hormones. CYPs are also essential for the conversion of environmental chemicals such as polycyclic aromatic hydrocarbons (PAHs) to carcinogenic metabolites that contribute to the genesis of breast cancer [15]. Critically, certain drugs used to treat advanced stages of breast cancer such as paclitaxel display little or no cytotoxic activity after metabolism by CYP2C8 and CYP3A4 (Figure 1) [16,17]. Figure 2 outlines drugs used clinically to treat various stages of breast cancer and which either benefit or suffer from intratumoural CYP expression [6,18].

In breast cancer research, there has been a variability between laboratories in identifying a particular CYP expression profile at both mRNA and protein levels. In a study conducted by Murray et al., the expression of a panel of 21 CYPs was studied in a cohort of 170 breast tumours by immunostaining of tissue microarray [19]. CYP2S1, CYP2U1 and CYP4X1 exhibited the highest percentage of strong immunoreactivity in contrast to other isoforms such as CYP2J and CYP3A4, which displayed no reactivity in majority of the tumours. A positive correlation with increasing tumour grade has been observed with the expression of CYP4V2, CYP4X1 and CYP4Z1; while CYP1B1, CYP3A5 and CYP51 were significantly associated with the ER status of the tumour. Among the 21 CYPs examined, absence of expression of CYP2S1, CYP3A4, CYP4V2 and CYP26A1 correlated with better survival even though none of them turned out to be an independent prognostic factor [19]. On a genetic level, a comparable study in a set of 20 paired samples of tumour and adjacent normal breast tissues from patients with infiltrating ductal carcinoma identified the expression of *CYP1B1*, *CYP2B6*, *CYP2C*, *CYP2D6*, *CYP2E1*, *CYP4B1* and *CYP11A1* in both tumour and control tissues [15]. A very low level expression of *CYP2C9* and *CYP3A4* was observed by RT-PCR in a similar study conducted in a set of 40 breast cancer and adjacent normal tissues [20]. Nevertheless, *CYP1B1* was overexpressed in one third of the tumours with a mean value exceeding a 50-fold increase compared to *CYP2E1*. Significantly higher expression of *CYP2E1* was found to be correlated with an invasive lobular tumour type and advanced disease compared to the invasive ductal ones, suggesting its potential role as a prognosis marker [20]. Some of these CYPs have been extensively studied on account of their multifactorial roles in breast cancer aetiology and treatment. The purpose of this review is to provide an update on how the presence of CYPs impacts on standard of care (SoC) drugs used to treat breast cancer as well as discuss opportunities to exploit CYP regulation and/or function for therapeutic intervention.

### 3.1. CYP1A1/A2

Expression of CYP1A1 is induced through the binding of substrates to the cytosolic aryl hydrocarbon receptor (AhR) [21]. Elevated *CYP1A1* mRNA expression has been detected to varying degrees in many breast cancer subtypes which supports its potential as a drug target in breast cancer treatment [22]. Expression of CYP1A1 has been found to be high in breast tumour cells with a positive correlation to tumour grade and menopausal status in newly diagnosed patients with adenocarcinoma of the breast [23]. Additionally, it has been found to be overexpressed in breast cancers that are resistant to anti-oestrogen treatment [24]. Knockdown of *CYP1A1* has been shown to impair proliferation and survival in the breast cancer cell lines MCF-7 and MDA-MB-231 through activation of AMPK signaling and inhibition of the phosphorylation of AKT, ERK and P70S6K [22]. There is also some evidence that CYP1A1 is important for maintaining the integrity of breast cancer stem cells (BCSCs), possibly by acting through β-catenin and PTEN/AKT signaling [25]. Increased basal level expression of CYP1A1 has also been observed in spheroids cultured from MCF-7 breast cancer cells, compared to the monolayer, which was suppressed upon co-culture with CD14^+^ cells in a macrophage-shaped environment [26]. This observation reinforces the suppressive effect of inflammatory conditions on the expression of CYP1A1 in mammary epithelial cells which is an important factor that has to be considered while designing immunomodulatory compounds and other chemotherapeutics that are bioactivated by CYP1A1.

Several studies have shown that polymorphisms in drug-metabolising genes not only pose as a risk factor for malignancy but also can serve as a predictive marker for drug efficacy and targeted therapies [27,28]. In metastatic breast cancer, patients with the CYP1A1*2C gene variant responded better to a treatment regimen involving docetaxel and capecitabine compared to those with the homozygous wildtype (CYP1A1*1) genotype (AA) who experienced a better efficacy with a combination of docetaxel with thiotepa [29]. In early breast cancer, *CYP1A2* rs762551 has been shown to be a potential predictive marker for patients treated with aromatase inhibitors paving the way towards individualised tailored assessment of therapy [30].

**Figure 3:**
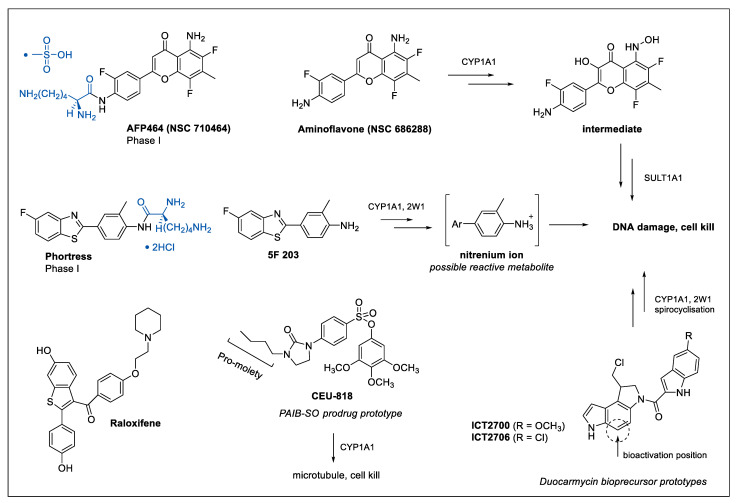
Prodrugs and bioprecursors targeting CYP1A1 or CYP1A1/2W1 for tumour-selective bioactivation.

Prodrugs that are bioactivated by CYP1 enzymes can broadly be classified into four groups of agents and include flavonoids, benzothiazoles, stilbenes and alkylating agents [29]. Aminoflavone (NSC686288, Figure 3), a ligand of AhR, has been shown to selectively induce apoptosis in MCF-7 breast cancer cells by activating the transcription of *CYP1A1* and *CYP1B1* with functional activity confirmed using the ethoxyresorufin-O-deethylase (EROD) activity assay both in vitro and in xenograft models [31]. Specific activation of the lysine prodrug of aminoflavone (AFP464, NSC710464) and subsequent CYP1 metabolic activation has been observed to be particularly effective in ER-positive breast cancer cells compared with ER-negative counterparts. Epigenetic reactivation of ERα expression treatment with the histone deacetylase (HDAC) inhibitor vorinostat has been shown to sensitise resistant cells towards AFP464, through the transcriptional induction of CYP1A1 [32]. Furthermore, AFP464 also inhibits the growth of tamoxifen-resistant breast cancer cells exhibiting increased expression of α6-integrin linked to suppression of the α6-integrin-Src-Akt signaling pathway [32]. Tamoxifen is a selective oestrogen receptor modulator (SERM) used in the treatment of hormone-receptor-positive breast cancers. AF464 entered a phase 1 clinical trial for advanced solid tumours where a maximally tolerated dose (MTD) of 54 mg/m^2^ on day 1, 8 and 15 of a 28-day cycle and dose-limiting pulmonary toxicity was observed [33]. Another prodrug, phortress, with selectivity to ER-positive cells, has also been shown to be an AhR ligand, effectively inducing CYP1A1 via the induction of the metabolite 5F203 to generate a number of metabolites including a presumed nitrenium ion intermediate capable of causing DNA damage (Figure 3) [34]. More recently, CYP2W1 has also been shown to contribute to the bioactivation of 5F203 [35]. In a phase 1 study, no MTD was determined for phortress while pulmonary and liver toxicities were observed [36]. The 50 patients that received phortress had advanced disease including bowel, lung, oesophageal and stomach cancer, and the study concluded that a “lack of efficacy” was observed, with no further clinical studies conducted. However, in preclinical studies, phortress was first and foremost being progressed as a prodrug with potential in ovarian and breast cancer but none of the 50 patients in the phase I trial harboured these cancers, meaning the true efficacy remains unknown [34,37]. Despite the discontinued clinical studies of AFP464 and phortress, AhR remains an interesting target for 5F 203 [38] and several promising preclinical strategies have been discussed in a recent review by McCluskey and co-workers and hence we refer to this for acquiring further knowledge [32].

Bioprecursor and prodrug approaches that rely on tumour-expressed CYP1A1 and not AhR induction for bioactivation includes our own work focused on reengineered duocarmycin molecules [39]. Duocarmycins are a class of exquisitely potent naturally occurring anti-tumour antibiotics that covalently bind the N3-position of adenine in the minor groove of DNA, subsequently resulting in cell death [40]. The clinical use of these compounds as anticancer agents is limited due to lack of tumour selectivity [41], however deactivation of the pharmacophore offers an opportunity to selectively target the oxidative capacity of CYP enzymes overexpressed in tumour tissue to restore the cell-killing ability of these compounds [42,43]. The xenograft proof-of-concept studies have been successful in bladder [44] and colorectal cancer [45] while preliminary data suggest the approach can be translated to breast cancer as well [39]. Patterson and co-workers have also shown that CYP1A1, as one of several CYPs, contributes in the conversion of the hypoxia-activated prodrug AQ4N to the topoisomerase II inhibitor AQ4 (Figure 2D), which has shown promise in a phase I trial that included six breast cancer patients [46,47]. Finally, CYP1A2 has been shown to contribute to bioactivation of the prodrug Tegafur to 5-FU [48], which is frequently used as a part of postoperative combination treatment that include tegafur–uracil (UFT) and tegafur–gimeracil–oteracil potassium (TS-1).

Another class of agent, the phenyl 4-(2-oxo-3-alkylimidazolidin-1-yl) benzenesulfonates (PAIB-SOs), has recently been reported as CYP1A1-targeting prodrugs of anti-microtubule-binding cytotoxins. The PAIB-SOs have been shown to display potent cytotoxic activity in both CYP1A1-expressing hormone-dependent and chemo-resistant breast cancers cells both in vitro and in vivo via *N*-dealkylation and prodrug activation (Figure 3) [49].

### 3.2. CYP1B1

Extrahepatic enzyme CYP1B1 has approximately 40% homology with CYP1A1 or CYP1A2 and plays a vital role in the treatment of receptor-positive breast cancers due to its ability to metabolise steroid hormones [12,50]. Expression of CYP1B1 is induced through the AhR receptor by ligands such as polycyclic hydrocarbons and dioxins akin to CYP1As and is mainly observed in tissues dependent on hormones such as mammary gland, ovary and uterus. *CYP1B1* is the most frequently expressed gene in breast cancer among the CYP1 family members [51]. However, there have been conflicting reports regarding the expression of CYP1B1 in tumours vs. the adjacent non tumour mammary gland tissues [20]. Although one reason for this disparity could be the increased sensitivity of transcript detection compared to protein, the lower expression of *CYP1B1* in cancerous tissue relative to adjacent could also be attributed to the possible downregulation of the gene by the AhR repressor in the tumour or by methylation of the promoter region, resulting in epigenetic silencing [52]. Nonetheless, expression of CYP1B1 in hormone-mediated malignancies is acknowledged to be important in the regulation of progression, metabolism, treatment and resistance of breast tumours [53]. Along with inactivating clinically relevant anticancer agents such as docetaxel and tamoxifen, CYP1B1 has also been shown to metabolise tamoxifen, 17 β-estradiol and bioactivate the natural product resveratrol to piceatannol, a metabolite with antiproliferative activity [54]. Single nucleotide polymorphisms (SNPs) have been found in the *CYP1B1* gene, which accounts for the variation of drug efficacy and toxicity between individual patients. A recent study reported that the expression of *CYP1B1* and SNPs 142 C > G, 4326 C > G were observed in TNBC patients with a higher rate of metastasis and recurrence following administration with taxane, adriamycin and cyclophosphamide (TAC) chemotherapeutic regimen [52]. Moreover, a higher expression of CYP1B1 was observed to be associated with increased drug resistance in breast cancer cells corroborating its role as a predictor of drug resistance and a prognostic indicator of treatment [55]. An overexpression of CYP1B1 and AhR has also been observed in inflammatory breast cancer (IBC) which correlated significantly with tumour grade, lymphovascular invasion, metastatic lymph nodes and the expression of Ki67 indicating cell proliferation [56]. Inhibiting the expression of AhR using CRISPR-Cas9 resulted in the decreased expression of CYP1B1, Wnt5a/b, and β-catenin in the IBC cell line SUM 149 [56]. Flow cytometry analysis also revealed that the subset of cells expressing a CSC phenotype (CD44+ CD24-/low) was positively correlated with the expression of AhR/CYP1B1, Wnt5a/b and β-catenin in the carcinoma tissues of IBC patients. This reinstated the role of AhR signaling in maintaining BCSCs subpopulations [25,56,57]. Whilst this study did not provide a clear understanding of the potential role of other AhR-induced CYP1 isoforms in breast CSCs, it partially substantiates the involvement of the receptor in regulating the stem cell compartment within the tumour microenvironment.

### 3.3. CYP2A6/ CYP2A7

CYP2A6 is primarily involved in the metabolism of nicotine and hence is associated with tobacco-related disorders including lung cancer. It is also one of the multiple CYPs involved in the metabolism of tamoxifen (Figure 2A). In a study by Bieche et al., *CYP2A6* was identified as one among seven genes coding for major xenobiotic-metabolising enzymes involved in breast tumourigenesis and which displayed a higher expression in ER-α-positive breast tumours compared with ER-α-negative tumours and normal mammary tissues [58]. A 25% upregulation in *CYP2A6* expression has been observed among ER-α-positive post-menopausal breast cancer patients, which is contradictory to a study conducted by Iscan and colleagues that failed to detect any mRNA expression of *CYP2A6* in healthy or cancerous breast tissues [15]. A possible explanation for these contradictory results may be due to the fact that the latter study was conducted only in infiltrating ductal carcinoma subtype of breast cancer without considering the hormone receptor status of the patients and also had a limited sample size. Clearly, a more exact understanding of CYP2A6 expression is significant for those patients who are being administered the prodrugs Tegafur and cyclophosphamide (CPA) [14,59] that in part rely on CYP2A6 bioactivation (Figure 2B,C).

### 3.4. CYP2A13/2B6

In addition to CYP2A6, CYP2A13 also plays a significant role in smoking-induced lung cancer due to its involvement in metabolising tobacco-specific nitrosamines [60]. In breast cancer, mRNA expression of *CYP2B6* has been detected in both healthy and cancerous breast tissues [15]. In pre-menopausal women with ER- and/or PR-positive breast cancer, SNPs in *CYP2B6* such as *CYP2B6* rs4802101 (T/T) and *CYP2B6* rs3211371 (T/C) have been associated with adverse prognosis [61]. The variability in the expression and function of this isoform between individuals stem from its inducibility and frequent polymorphism, respectively. CYP2B6 also plays major role in activating CPA in the liver by oxidising the prodrug to a 4-hydroxycyclophosphamide (4-OH-CPA) which subsequently produces a cytotoxic alkylating agent that damages DNA and eradicates tumour cells [14] (Figure 2C). SNPs in *CYB2B6* can have considerable impact on the expression, function and pharmacokinetics of CPA. Variation in the alleles of *CYP2B6* such as *CYP2B6* *2, *4, *8 and *9 have been correlated with poor outcome while *CYP2B6* *5 has been associated with longer progression-free survival and higher dose delay incidence [62]. In metastatic breast cancer patients receiving a combination of the chemotherapeutic drugs docetaxel and thiotepa, it was observed that altered *CYP2B6* genotypes such as AG genotype of rs2279343 and a CT/TT genotype of rs8192719 were beneficial and that the former was associated with a decreased death risk (47%) and an overall survival increase of 6 months among patients with liver metastasis [63]. Moreover, an in vitro study has shown 17 β-estradiol to upregulate the expression of CYP2B6 exclusively in T-47D but not in MCF-7 breast cancer cells by increasing the recruitment of ER-α and nuclear receptor coactivator 3 (NCoA3) to the 5’ flanking region of the enzyme [64]. ER binding sites were also observed in an upstream 5’ regulatory region of the *CYP2B6* gene in several breast cancer cell lines.

### 3.5. CYP2C 8/9/18/19

Among all enzymes belonging to the CYP2C family, which contribute around 20% of the total CYP transcript expression in human liver, *CYP2C9* is the most abundant. CYP2C enzymes are known to contribute to the activation of prodrugs such as the oxazaphosphorines [65], including CPA shown in Figure 2C. Both RNA and protein-level expression of CYP2C9 and gene-level expression of *CYP2C8* have been reported in a small cohort of 10 breast tumour samples [66]. No expression of *CYP2C18* and *CYP2C19* was observed in these samples, which could be attributed to the downregulation of these CYPs by IL-6 cytokines in an autocrine manner, similar to observations in liver tissues [66]. Among genetic variants of these genes, CYP2C9*2 polymorphism was found to be significantly correlated with inferior therapeutic benefit from neoadjuvant chemotherapy [67]. Similarly, the efficacy of tamoxifen treatment in ER-positive breast cancer patients might be influenced by an rs4917623 SNP in the *CYP2C19* gene [68].

### 3.6. CYP2D6

Bioactivation of tamoxifen to a more potent, pharmacologically active and abundant anti-oestrogenic metabolite “endoxifen” (4-hydroxy-*N*-desmethyltamoxifen) is a two-stage process that involves *N*-desmethylation and 4-hydroxylation steps, which depends mainly on the enzymatic activity of the rate-limiting enzyme CYP2D6 (Figure 2A). As a result, the clinical efficacy of tamoxifen in each patient is dependent on the concentration of endoxifen which further relies on the activity of the highly polymorphic enzyme CYP2D6 [69,70]. This makes polymorphisms a key predictor of tamoxifen outcome in breast cancer patients [71]. Genetic variants of CYP2D6 can be classified based on the activity of the enzyme as extensive, intermediate and poor metabolisers and these genotypes have a gene-dose effect on the endoxifen concentrations in patients treated with tamoxifen [72]. In a study conducted by Blancas et al. in 2018, a significantly lower disease-free survival was observed in a set of 87 patients taking adjuvant tamoxifen treatment with slow metabolisers compared to the rapid counterparts [73]; we refer the reader to in-depth reviews [74,75] of how the variant alleles of *CYP2D6* can be used to predict the outcome in tamoxifen-treated breast cancer patients. A recent study conducted with data from Swedish breast cancer cohorts demonstrated a worse prognosis for both rapid and slow metabolisers (compared to normal), which could be due to the occurrence of adverse effects of the treatment [76].

### 3.7. CYP2E1

Apart from being a phase 1 drug-metabolising enzyme, CYP2E1 is also associated with several diseases like obesity, type-2 diabetes and liver diseases related to alcohol consumption and cancer [77]. Increased expression of CYP2E1 in breast tumours correlated with more serious and invasive lobular types of tumours [20]. Ectopic expression of the enzyme in breast cancer cells has been shown to induce oxidative and ER stress, autophagy while an inhibitory effect on metastasis was modulated by p53 as a possible upstream regulator [77]. Furthermore, evidence also suggests that CYP2E1 regulates cellular energy metabolism in a manner dependent on cell type and potentially on the clinical staging of the disease and suggest a possible biomarker role [78]. The genetic polymorphic variant CYP2E1 *6 *Dra1 (D/C)* has also been shown to correlate with higher risk of developing breast cancer and warrants further investigation [79].

### 3.8. CYP2S1 and CYP2W1

Known as orphan CYPs, the biological functions of dioxin-inducible CYP2S1 and CYP2W1 have not been elucidated [80]. Both isoforms appear to be differentially expressed in cancerous tissues and their orphan status makes it interesting to explore their potential as therapeutic targets. In a similar manner to CYP1 family members, AhR and ARNT regulate the induction of CYP2S1 and the enzyme has been linked to metabolism of toxic carcinogenic compounds via the peroxidase shunt [80,81]. The expression of CYP2S1 is distributed among a variety of extrahepatic epithelial tissues that are susceptible to the exposure of carcinogens and xenobiotics and also in several epithelial-derived cancers including breast [19,80]. Contrarily, *CYP2W1* transcripts are mainly restricted to foetal and tumour tissues with minimal extrahepatic expression [82,83]. Apart from metabolising procarcinogens such as PAHs, CYP2W1 is also involved in the oxidation of indoline substrates, lysophospholipids, free fatty acids and 17 β-estradiol [83]. Hlavac et al. in 2014 assessed transcript levels of 10 CYP enzymes in 68 post treatment tumours of breast carcinoma patients and reported significantly higher intratumoural CYP2W1 in responders of neoadjuvant chemotherapy compared to non- responders [84]. In breast cancer cell lines MCF-7 and MDA-MB-231, constitutive expression of both CYP2S1 and CYP2W1were detected, with greater abundance in MDA-MB-231 cells [82]. Moreover, treating the cells with methoxy stilbenes or resveratrol increased the expression of *CYP2S1* and *CYP2W1* in MCF-7 but not in MDA-MB-231 cells at the genetic level. The benzothiazole 5F203 (and analogue GW-610) was found to selectively induce the expression of CYP2S1 and CYP2W1 in isogenic breast cancer cells depleted for the isoforms [85]. Gene knockdown studies in breast cancer cells identified the role of CYP2S1 in metabolically inactivating benzothiazoles while induction of CYP1A1 remained crucial for their activation and anti-tumour properties [85]. In the molecular taxonomy of breast cancer international consortium (METABRIC) cohort, high *CYP2S1* mRNA was significantly associated with basal like breast cancers compared to *CYP2W1* which was predominantly expressed in luminal A tumours (SM and RA unpublished data).

### 3.9. CYP3A4/A5

CYP3A4 is abundantly expressed and plays major role in metabolising 60% of the marketed drugs including chemotherapy used to treat breast cancer patients such as CPA, docetaxel, paclitaxel, etoposide, irinotecan, imatinib, lapatinib, trastuzumab emtansine and tamoxifen [18,86]. CYP3A5 is commonly expressed in HER-2-positive breast cancers [84] and is reported to be involved in the bioactivation of lapatinib [87]. Lapatinib, used in the treatment of HER-2-positive metastatic breast cancer is metabolised by CYP3A4/5 in the liver. The metabolites have been known to cause idiosyncratic hepatocellular toxicity [88]. In addition, the nitroso metabolites of lapatinib can bind CYP3A4 forming a metabolite–inhibitor complex that can inactivate CYP3A4 [89], however the clinical consequences of the latter is unknown. Tumours negative for the expression of CYP3A4 have been shown to have a better response rate to the antineoplastic drug docetaxel than those expressing the enzyme [12]. Furthermore, higher expression of CYP3A4 has been shown to be related to tumour progression in ER+ breast cancer cells, in part via the synthesis of epoxyeicosatrienoic acids [90]. In 2001, Schmidt and colleagues identified the presence of CYP3A4 in breast cancer microsomes which aided the metabolism and activation of the CPA prodrug analogue ifosfamide [91]. DMI—a component of the trastuzumab–DMI conjugate used in the treatment of high-risk early stage and metastatic HER-2-positive breast cancer [92], is metabolised by CYP3A4 and to a lesser extent by CYP3A5. Administering trastuzumab–DMI with other agents that can act as CYP3A4 inhibitors (e.g., atazanavir, clarithromycin, ketoconazole and indinavir) should be avoided to minimise potential drug-related toxicity.

In postmenopausal tamoxifen-treated breast cancer patients, a genetic polymorphism in *CYP3A5* (CYP3A5*3) was found to be correlated with a positive response [93]. This finding is contrary to that of Spitman et al. who suggested that there is no added benefit of *CYP3A* genotype additionally to *CYP2D6* variants on tamoxifen metabolism [94]. As the authors claim, this discrepancy could possibly be due to the use of CYP3A4/5 inhibitors during the latter study, which were not properly evaluated owing to the minimal data availability. However, the mechanisms leading to tamoxifen resistance are less likely to be caused by a single gene polymorphism since the metabolism of the drug is complex involving several CYP enzymes.

### 3.10. CYP4Z1/Z2P

Orphan cytochrome CYP4Z1 is also known as a breast cancer-associated cytochrome due to its putative role in breast cancer through the formation of a signaling molecule 20-hydroxyeicosatetraenoic acid (20-HETE) [95]. *CYP4Z1* and its transcribed pseudogene *CYP4Z2P* (96% identity) were initially cloned from SKBr3 breast cancer cells. Both these enzymes are overexpressed in breast cancer, with CYP4Z2P having a similar expression pattern to CYP4Z1 but at a generally lower level which is likely due to the degraded non-functional protein product [96]. Expression of these enzymes is restricted to the mammary glands while they have been shown to be overexpressed in breast carcinoma tissues, which correlated with increasing tumour grade and poor prognosis [97]. Stable expression of CYP4Z1 in breast cancer cells has been shown to enhance angiogenesis and tumour growth in vivo by activating PI3K/Akt and ERK1/2 signaling pathways [97]. Intriguingly, in the breast cancer line MCF-7, CYP4Z1 was detected on the outside of the plasma membrane; however, no isotype-matched antibodies were included as negative controls and so the results must be treated with caution [98]. The study also detected autoantibodies against the enzyme in the sera of patients with breast cancer in comparison to the control samples suggesting a prospective role as a tumour-associated antigen [98]. The miRNA-mediated competing endogenous RNA network (ceRNET) between *CYP4Z1* and *CYP4Z2P* has been suggested to inhibit apoptosis in breast tumours by serving as a sub-ceRNET to *TERT* and also promotes angiogenesis and CDK3 expression [99].

### 3.11. CYP17A1 and CYP19A1

Endocrine therapy includes the use of SERMs such as tamoxifen and raloxifene and the aromatase inhibitors: anastrozole, letrozole and examestane. The latter target CYP aromatase (CYP19A1, oestrogen synthase) by intervening in the synthesis of aromatic C18 oestrogens (oestrone and oestradiol) from C19 androgens (androstenedione and testosterone). Mechanistic insights into CYP19A1 function, polymorphism and implication have recently been reported and we refer to a recent review for further reading [100].

Although the steroidogenic biosynthetic pathway is complex and multifactorial, it does offer targets for therapeutic intervention [101]. In men, CYP17A1 is a successful target for abiraterone treatment of patients diagnosed with castration-resistant prostate cancer. Abiraterone has also been explored in the treatment of patients with ER+ metastatic breast cancer, as a single agent or in combination with exemestane but with no improvement in progression-free survival [102]. Nonetheless, it is possible that a CYP17A1-targeting agent such as abiraterone could be used with benefit in a subset of TNBC patients harbouring tumours no longer responsive to standard treatment [103].

### 3.12. CYP24

The mitochondrial enzyme CYP24A1 (24-hydroxylase) manifests its key role in inactivating 1 α, 25-dihydroxyvitamin D_3_ (1,25-(OH)_2_D_3_), the biologically active form of the secosteroid hormone vitamin D and is a candidate oncogene [104]. 1,25-(OH)_2_D_3_ binds to the vitamin D receptor (VDR) which is a ligand-activated nuclear receptor, which activates a network of genes that regulate calcium homeostasis, cell growth, differentiation and functional activities of different cell types [105]. Elevated expression of *CYP24A1*, an immediate target gene of VDR, has been observed in breast cancer cells with aberrant amplification of the chromosomal loci encoding the gene [106,107]. In the absence of ligand, the basal mRNA expression of *CYP24A1* correlated inversely with VDR protein levels in several breast cancer cell lines and the unliganded VDR exerts a repressive action on CYP24A1 [108]. Knockdown of *CYP24A1* using shRNA in mammary tumour lines resulted in the suppression of the metabolism of vitamin D and abrogated tumour growth in vivo, supporting the hypothesis that deficiency of vitamin D could be related to breast cancer incidence [109]. There are several inhibitors for CYP24A1 including the naturally occurring soy-derived isoflavane genestein which inhibits both CYP24A1 and CYP27B1 in a time- and dose-dependent manner; the latter is an enzyme that also catalyses the conversion of 25-hydroxyvitamin D_3_ to 1,25-D_3_ [106,110].

### 3.13. CYP26A1/B1

CYP26 family generally functions by metabolising and clearing all-*trans*-retinoic acid (ATRA) which is an active metabolite of vitamin A or retinol, involved in regulating cellular differentiation, proliferation, migration and apoptosis [111]. The major role of the highly conserved members of this family CYP26A1, CYP26B1 and CYP26C1 appears to be in the conversion of retinoic acid (RA) to its metabolites, primarily 4-OH-RA. Upregulation or overexpression of CYP26A1 triggers the signals for cell survival and anti-apoptosis by inducing oncogenes and downregulating tumour suppressor genes along with deregulating the cell cycle and DNA repair genes, perhaps indicating properties akin to an oncogene [112,113]. CYP26A1 has been shown to be expressed at a constitutively higher level in breast adenocarcinomas which can be selectively inhibited, e.g., by use of a RA metabolism blocking agent R116010, demonstrating an anti-tumour effect in ER+ mouse tumours [106]. Osanai et al. have demonstrated a strong cytoplasmic expression of CYP26A1 in 42% of the primary breast carcinomas samples by immunohistochemistry with significantly more intense staining observed in metastatic tumours. Breast cancer cells overexpressing *CYP26A1* displayed oncogenic properties, improved cell survival, higher resistance to anoikis and enhanced tumorigenicity and metastatic potential [113]. RA metabolism blocking agents (RAMBAs) together with ATRA have been shown to exhibit a synergistic effect in regressing ER+ MCF-7 and MCF-7Ca xenografts in nude mice [114]. CYP26A1 also regulates the expression of the actin-bundling protein fascin by modulating the levels of RA thereby affecting the malignant behaviour of CYP26A1 expressing breast cancer cells [115].

### 3.14. CYP27A1

As a part of regulating intracellular cholesterol homeostasis, CYP27A1 (27-hydroxylase) catalyses the hydroxylation of cholesterol to an oxysterol known as 27-hydroxycholesterol (27HC), which is a SERM that can act as a partial agonist of the ER in breast cancers subsequently stimulating growth and metastasis [116]. Differential expression of CYP27A1 has been observed in breast tumours correlating with the phenotype and prognosis of the disease [117]. The role of CYP27A1 in metastasis is mediated by the action of circulating metabolite 27HC on the function of myeloid cells located at distant metastatic niche inducing a suppressed immune microenvironment [118,119]. Treatment with statins, drugs used to manage hypercholesterolemia, have been shown to reduce the concentration of 27HC in the serum together with deregulation of the expression of this enzyme in breast tumours [117]. Inhibition of CYP27A1 with aromatase inhibitors, such anastrozole and fradrozole, has also proved to be beneficial in treating breast tumours dependent on ER signaling [120].

## 4. CYP Isoform Expression in the Distinct Breast Cancer Subtypes and Implication in Patient Survival

**Figure 4:**
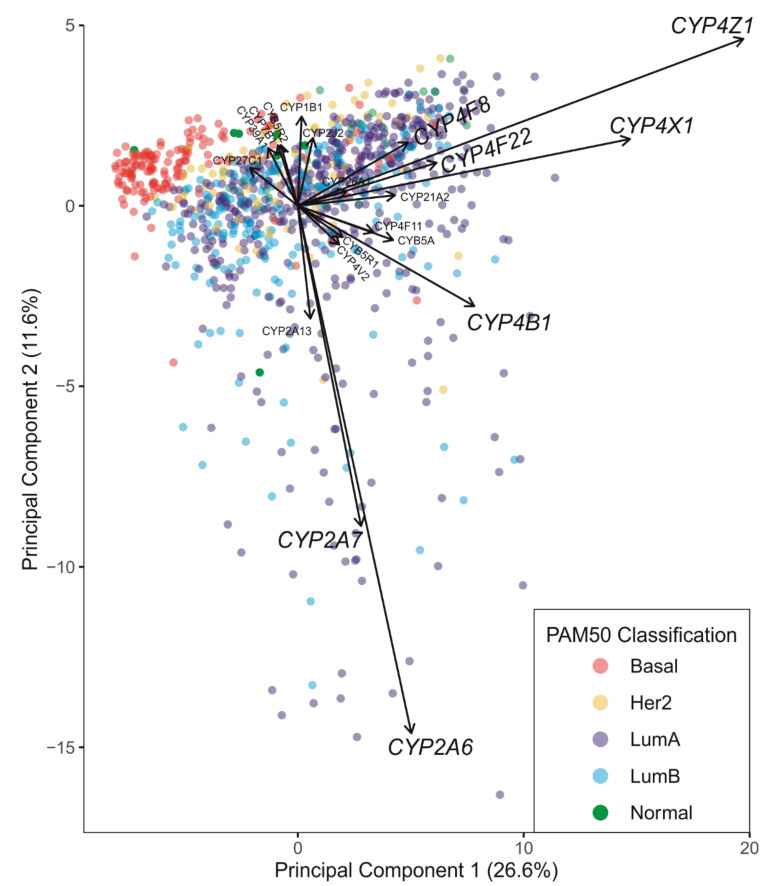
Variance in cytochrome P450 (*CYP*) gene expression (and their essential reductases) was analysed by principal component analysis (PCA). In the PCA biplot, each point represents an individual tumour from The Cancer Genome Atlas (TCGA) breast cancer cohort (*n* = 935) coloured according to the PAM50 classification [122]. The size and direction of arrows assigned to the top 20 most variant genes in the cohort represent the PCA loadings and reflect how strongly each gene influences principal components 1 and 2.

The impact of pharmacogenomics on cancer treatment together with our increasing understanding of the role and presence of drug-metabolising enzymes supports the choice of SoC drugs as well as in the discovery of new therapies. In breast cancer, a more routine molecular profiling will allow the assessment of CYP expression patterns in support of patient treatment selection, e.g., tamoxifen is mostly effective in patients expressing CYP2D6 while in contrast paclitaxel is largely ineffective in patients expressing high levels of CYP2C8 and CYP3A4. Design of targeted approaches exploiting the presence of selective CYPs is key for tumour-selective therapy with minimised side effects. This is relevant in neoadjuvant or adjuvant settings where conventional chemotherapeutic regimens are used to reduce tumour bulk and/or growth and metastasis. However, certain factors should be taken into consideration while utilising pharmacogenetics in personalising treatment. Data from several studies suggests that multiple drug-metabolising genes are involved in the metabolism of anticancer agents and signaling of oestrogen receptors. In this regard, profiling of CYPs prior to treatment will support clinicians in predicting drug efficacy and treatment outcomes. Moreover, there are possibilities of other accompanying medications which can inhibit CYP enzymes thereby altering the drug metabolism and outcome [121]. Discontinuation of a therapy is another variable which is pivotal as a poor metaboliser may benefit more from a continuous treatment than an extensive metaboliser who discontinues the medication due to its side effects. Despite these shortcomings, tailoring patient-specific CYP-directed therapeutic regimens in combination with routine chemotherapeutic agents is a promising way to manage breast cancer. Interestingly, despite the vast number of papers involving CYP expression in breast cancer, sparse information is available on CYP isoform expression in the distinct breast cancer subtypes. Principal component analysis using The Cancer Genome Atlas Breast Invasive Carcinoma (TCGA)-BRCA mRNAseq transcriptomic counts data (*n* = 935) with PAM50 subtype classifications (“Basal-like”, “Her2-enriched”, “Luminal A”, “Luminal B”, “Normal-like”) [122] revealed the CYP4 members *CYP4X1* and *CYP4Z1* to be highly expressed but not variable in a way that is not associated with the PAM50 classifications (Figure 4 and Figure S1). *CYP2A6*, *CYP2B6*, *CYP2D6*, *CYP3A4* and *CYP3A5* involved in drug metabolism of the breast cancer prodrugs CPA, tegafur and tamoxifen (Figure 3) were expressed at various levels, but no significant differences between the PAM50 groups were observed (data not shown). The divergence of CYPs expression variance from the PAM50 classifications is concerning because it means they will require separate analysis to form a distinct CYP profile. Currently we have only a limited basis for the interpretation of a molecular CYP profile; however, we hope the collection of these data will lead to enhanced therapeutic efficacy. Care must be taken with interpretation of any molecular CYP profile in light of the transient nature of CYP expression, their rapid response to any ongoing treatment will make rigorous collection of metadata critical to objective assessment.

**Figure 5:**
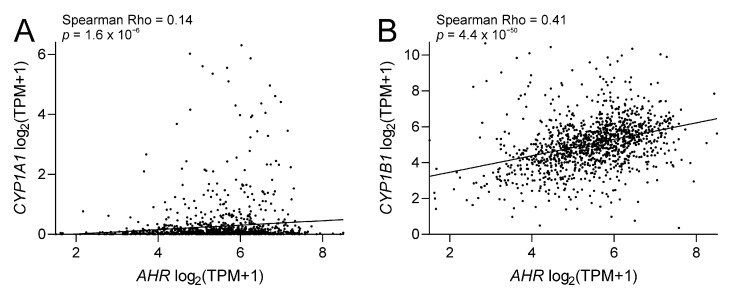
Scatter plots showing the relationship between aryl hydrocarbon receptor (*AHR*) and *CYP1A1* (**A**) or *CYP1B1* (**B**) gene expression, found both *CYPs* were significantly correlated with *AHR* in TCGA breast tumours (*n* = 1222).

CYP1 analysis of TCGA-BRCA RNAseq data revealed *CYP1A2* expression to be very low (max TPM = 1.3 with a median TPM = 0), while expression of *CYP1A1* was high in a subset of tumours (max TPM = 78.3 but median TPM = 0.04) and *CYP1B1* was generally more abundant (max TPM = 1611.7 and median TPM = 31.9). Both *CYP1A1* and *CYP1B1* showed significant (*p* < 0.0001) correlation with *AhR* expression (Spearman Rho = 0.14 and 0.41, respectively). Interestingly, the analysis indicates that *CYP1B1* is likely regulated by two different mechanisms because basal *CYP1B1* expression is retained even in *AhR*-low tumours, a feature that is not evident for *CYP1A1* (Figure 5). CYP1B1 is implicated in drug sensitivity so this observation is important for treatment-naïve patients. Future studies are needed to evaluate transcript data from patients who have received AI or chemotherapy treatment and it is possible much higher expression of CYPs induced by AhR, CAR and PXR would be noticeable and an important factor to consider in patients with relapsed tumours.

The METABRIC analysis of CYP genes involved in the metabolism of anti-tumour agents in breast cancer unravelled the association of some of these isoforms with patient survival. In total, mRNA expression of 24 CYP genes was analysed using the METABRIC cohort, of which expression of 9 genes were significant and expression of 15 were not significant with respect to patient survival.

**Figure 6:**
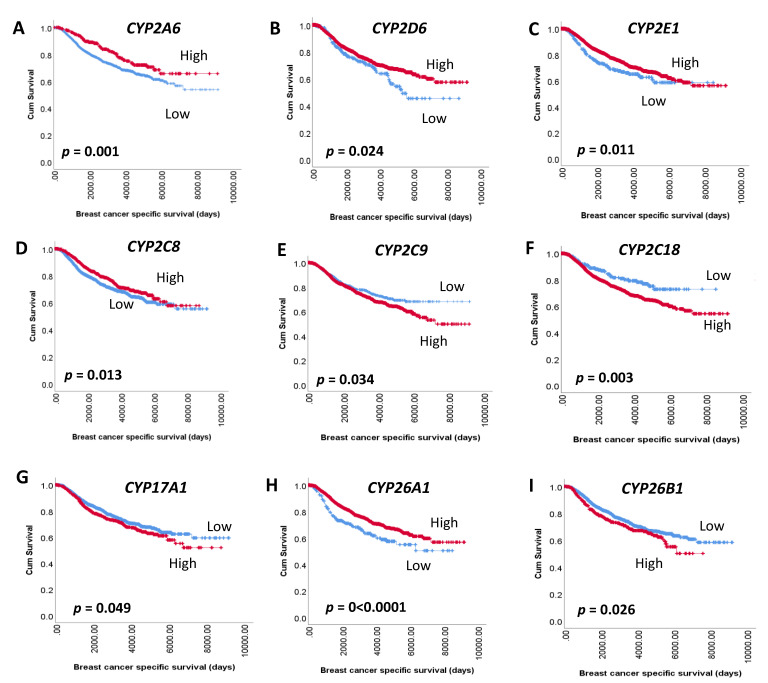
Kaplan–Meier survival analysis of CYP mRNA on breast cancer-specific survival. Survival curves showing the impact of low (blue line) and high (red line) mRNA expression: (**A**) *CYP2A6* (**B**) *CYP2D6* (**C**) *CYP2E1* (**D**) *CYP2C8* (**E**) *CYP2C9* (**F**) *CYP2C18* (**G**) *CYP17A1* (**H**) *CYP26A1* (**I**) *CYP26B1*. Significance was determined using the log-rank test. Significant *p*-values (<0.05) are indicated in bold.

Kaplan Meier survival analysis shows that low *CYP2A6* (*p* = 0.001), *CYP2D6* (*p* = 0.024), *CYP2C8* (*p* = 0.013), *CYP2E1* (*p* = 0.011), *CYP26A1* (*p* < 0.0001) and high *CYP2C9* (*p* = 0.034), *CYP2C18* (*p* = 0.003), *CYP26B1* (*p* = 0.026) and *CYP17A1* (*p* = 0.049) mRNA expression, in the total patient cohort, was significantly associated with adverse breast cancer-specific survival (Figure 6). Overall, 434/1603 (27%) patients with low *CYP2A6* expression died compared to 70/353 (19%) patients with high expression. A similar trend was observed for *CYP2D6* expression, wherein 68/220 (30%) patients with low expression had worse survival compared to 437/1738 (25%) patients with high expression. A proportion of 305/1125 (27%) patients with low *CYP2C8* expression had adverse survival compared to 200/834 (24%) with high expression. Similarly, low *CYP2E1* (85/281, 30%) and *CYP26A1* (87/251, 35%) expressing patients had adverse survival compared to those with high expression (420/1678, 25% and 418/1708, 24%, respectively).

An opposite trend was observed for *CYP2C9* with higher expression resulting in adverse survival, with 355/1297 (27%) events compared to 150/662 (22%) in patients with low *CYP2C9*. Similarly, patients with high *CYP2C18* (459/1698, 27%), *CYP26B1* (134/475, 28%) and *CYP17A1* (190/670, 28%) had worse survival compared to those with low expression (i.e., 46/261, 18%, 371/1484, 25% and 315/1289, 24%, respectively).

mRNA expression of *CYP1A1* (*p* = 0.123), *CYP1B1* (*p* = 0.109), *CYP2A13* (*p* = 0.195), CYP2C19 (*p* = 0.130), *CYP2S1* (*p* = 0.279), *CYP2W1* (*p* = 0.121), *CYP3A4* (*p*= 0.194), *CYP3A5* (*p* = 0.082), *CYP4Z1* (*p* = 0.113), *CYP4Z2P* (*p* = 0.092), *CYP19A1* (*p* = 0.054), *CYP24A1* (*p* = 0.285), *CYP26C1* (*p* = 0.055), *CYP27A1* (*p* = 0.074) and *CYP27B1* (*p* = 0.126), did not show any significant association with survival (data not shown). Survival analysis could not be conducted for *CYP1A2* or *CYP2B6* due to lack of gene expression information in the METABRIC dataset.

Lower expression of CYP2A6 and CYP2D6, involved in the metabolism of tamoxifen, tegafur and CPA [123,124] may result in inefficient metabolism/activation of these agents which may affect survival of breast cancer patients as demonstrated in the current study. Higher expression of CYP2C9 has been associated with cancer progression and aggressiveness with certain polymorphisms (CYP2C9*2 and CYP2C9*3) encoding enzymes with reduced activity [37]. Such polymorphic alleles may reduce responsiveness of patients to agents such as tamoxifen and CPA [125].

## 5. Discussion and Future Directions

In spite of the significant advances in treatment strategies over the past decade, selective treatment of breast cancer with limited off-target effects still remains a great challenge. CYP17A1 and CYP19A1 have been successfully pursued as targets for therapeutic intervention and small molecule-based drugs such as abiraterone and the AIs anastrozole, letrozole and exemastane are now established treatment options for prostate and breast cancer, respectively. Although not discovered through rationalised drug discovery, tamoxifen, tegafur and CPA are examples of CYP-activated prodrugs used to treat breast cancer. As these prodrugs are not bioactivated in a tumour-selective manner, administration of these agents is associated with side effects. Several prodrug strategies focused on targeting the AhR and CYP1A1 and CYP1B1 expression have not yet translated into drug approval for clinical use, but promising research in this area has been reported.

Considering the heterogeneity of breast tumours, it is well established that hypoxia plays a key part in both chemo- and radio-resistance. Hypoxia occurs in approximately 40% of breast cancers and is more commonly seen in locally advanced primary breast cancer [126]. Hypoxic regions have been found to occur in all breast cancer stages, from in situ carcinomas to both lobular and ductal invasive cancers [127]. Studies have reported that the mean pO_2_ in breast cancer ranges between 2.5 and 28mmHg (0.3–3.5% O_2_), with a median value of 10 mmHg (1.3% O_2_), in comparison to 65 mmHg (8% O_2_) in normal breast [128]. The presence of hypoxia in breast cancer decreases the effectiveness of hormonal therapy due to loss of ERα [129]. In patients with ductal carcinoma in situ (DCIS), the presence of hypoxia or necrosis is associated with a shorter time to recurrence [130]. Furthermore, the presence of hypoxia in primary breast cancers has been associated with hypoxia markers in their lymph node metastases, indicating that the hypoxia phenotype is maintained even in metastatic lesions [131]. Accordingly, the presence of hypoxia can be exploited for patient benefit via use of hypoxia-activated prodrug (HAP) therapy [132]. A rationalised drug design and understanding of the bioreductive capacity of CYPs [133] in solid tumours affected by hypoxic stress led to the development of AQ4N (Banoxantrone), which has reached clinical evaluation [47] but not yet market approval. AQ4N has been shown to be bioactivated under low oxygen tension by at least five CYPs (see Figure 2D) to generate AQ4, a potent topoisomerase II poison. Given that patients harbouring breast tumours with reduced oxygen tension have been linked to increased risk of mortality and metastasis [134], there is a real opportunity for use of HAPs as an adjuvant in combination with SoC treatment. Sadly, despite much promise, no HAP has yet been approved for clinical use [135] but this field warrants further investigation in defining predictive biomarkers for HAPs and overcoming the technical challenges of assaying them in clinical settings, as argued elegantly in a recent review by Wilson and co-workers [136].

Regardless, of inhibitor or prodrug approach relying on intratumoural CYP expression for therapeutic intervention, it is important to advance the understanding of CYP expression, function and regulation in the tumour microenvironment. This would include careful investigations of 2D and 3D breast cancer models including co-culture systems, spheroids, organoids, patient-derived xenograft models and clinical specimens. Analysis of data from TGCA database did not reveal clear expression differences associated with the PAM50 classification system as we have reported here, however more work is required to fully understand regulation and expression in treatment-naïve and differently treated patient cohorts. Furthermore, as alluded to in this review, it is also essential to take into consideration the presence of CYP polymorphic variants and their function to better understand how these impact on treatment outcomes.

In conclusion, acquiring in-depth knowledge on CYP expression and function is important for improving current SoC treatment as well as guiding new and ongoing efforts to develop drugs with improved therapeutic index for patient benefit.

## 6. Methods

### 6.1. TCGA Analysis

TCGA-BRCA mRNAseq transcriptomic counts data were downloaded from the National Cancer Institute (NCI) Genomic Data Commons dbGaP accession phs000178. The results published here are based upon data generated by The Cancer Genome Atlas (TCGA) managed by the NCI and National Human Genome Research Institute. Information about TCGA can be found at http://cancergenome.nih.gov (accessed 18 January 2019). PAM50 subtype classifications (“Basal-like”, “Her2-enriched”, “Luminal A”, “Luminal B”, “Normal-like”) were taken from Ciriello et al. [122]. TCGA-BRCA mRNAseq data were converted to transcripts per million (TPM) and log2 (TPM+1) values were used in principal component analysis (PCA). Values for protein-coding CYP genes were extracted and combined with those for the two essential reductases (P450 oxidoreductase: POR and cytochrome b5: CYB5A). Gene expression was analysed in an unscaled PCA using prcomp in R (version 3.6.1). Graphs were drawn by fviz_pca (version 1.0.3) in R. Plots display contributions of the top 20 genes for clarity. All other graphs and statistical analyses were generated in Prism (v.8.3.0; Graphpad).

### 6.2. METABRIC Analysis

Patient cohort

The molecular taxonomy of breast cancer international consortium (METABRIC) dataset, containing sequencing data of 1980 primary breast cancer patients [137], was used to assess the significance of CYP mRNA associations with patient survival. Information about the METABRIC data set is published in Curtis et al., 2012. Patient tumours were collected from 5 centres in the UK and Canada between 1977 and 2005. Median follow-up was 141 months. Patients who were ER-positive and/or lymph node-negative did not receive adjuvant chemotherapy, whereas all ER-negative and/or lymph node-positive patients were administered adjuvant therapy. mRNA was isolated from primary patient tumours in the METABRIC cohort and assayed using an Illumina HT-12 v3 platform [137].

### 6.3. Statistical Analysis

METABRIC cohort CYP data were stratified based on breast cancer-specific survival (BCSS) using X-Tile software version 3.6.1 [138]. To assess the relationship between mRNA expression and survival outcomes, survival curves were plotted using the Kaplan–Meier method with significance determined using the Log-rank test. A *p*-value of <0.05 was considered as statistically significant.

## Data Availability

Not applicable.

## Supplementary Materials

The following are available online at https://www.mdpi.com/2227-9059/9/3/290/s1, Figure S1: (A) CYP4Z1 and (B) CYP4X1 expression analysis using PAM50 classification.
