# Supplementary material for: Intratumoural Cytochrome P450 Expression in Breast Cancer: Impact on Standard of Care Treatment and New Efforts to Develop Tumour-Selective Therapies
Source: Biomedicines. 2021 Mar 12;9(3):290. doi: 10.3390/biomedicines9030290 (PMC7998590; doi:10.3390/biomedicines9030290)
Supplement: Supplementary file 1 [file biomedicines-09-00290-s001.pdf]

Supporting document

Intratumoural cytochrome P450 expression in breast cancer: impact on standard of care treatment and new efforts to develop tumour-selective therapies

Smarakan Sneha[1], Simon C. Baker[2], Andrew Green[3], Sarah Storr[3], Radhika Aiyappa[3], Stewart Martin[3] and Klaus Pors[1,*]

[1] Institute of Cancer Therapeutics, School of Pharmacy and Medical Sciences, Faculty of Life Sciences, University of Bradford, Bradford, BD7 1DP, UK; S.Swaroop@bradford.ac.uk

[2] Jack Birch Unit for Molecular Carcinogenesis, Department of Biology & York Biomedical Research Institute, University of York, Heslington, York YO10 5DD, UK; simon.baker@york.ac.uk

[3] Nottingham Breast Cancer Research Centre, School of Medicine, Biodiscovery Institute, University Park, Nottingham NG7 2RD, UK; andrew.green@nottingham.ac.uk (A.G.); sarah.storr@nottingham.ac.uk (S.S.); Radhika.Aiyappa@nottingham.ac.uk (R.A.); stewart.martin@nottingham.ac.uk (S.M.)

* Correspondence: k.pors1@bradford.ac.uk; Tel.: +44 (0)1274 236482(Office); +44 (0)1274 235866 (Lab); Fax: +44 (0)1274 233234

Corresponding author: k.pors1@bradford.ac.uk

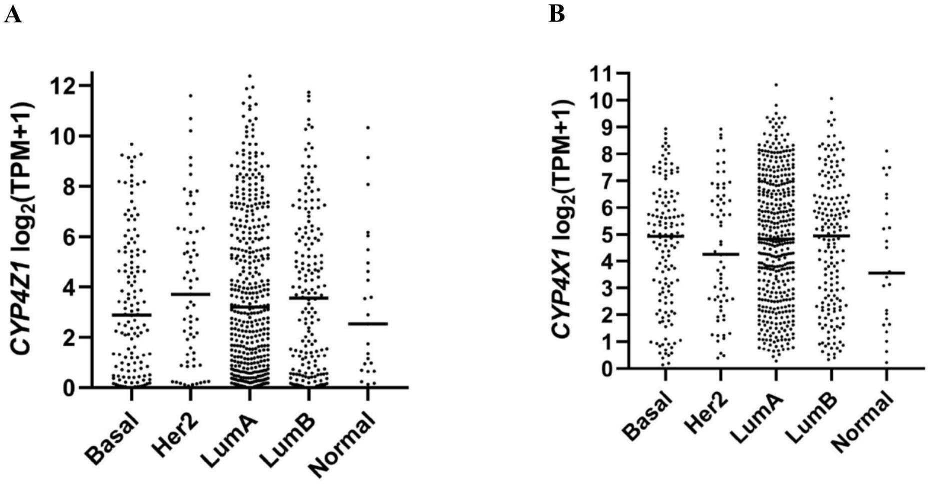

Figure S1. (A) CYP4Z1 and (B) CYP4X1 expression analysis using PAM50 classification. Variance in cytochrome P450 (*CYP*) gene expression (and their essential reductases) was analysed by principal component analysis (PCA). Details can be found in the methodology section in the main review paper
